# A New Trans-Tympanic Microphone Approach for Fully Implantable Hearing Devices

**DOI:** 10.3390/s150922798

**Published:** 2015-09-09

**Authors:** Seong Tak Woo, Dong Ho Shin, Hyung-Gyu Lim, Ki-Woong Seong, Peter Gottlieb, Sunil Puria, Kyu-Yup Lee, Jin-Ho Cho

**Affiliations:** 1 Graduate School of Electronic Engineering, Kyungpook National University, 80 Daehak-ro, Buk-gu, 41566 Daegu, Korea; E-Mails: biotak@knu.ac.kr (S.T.W.); swap9552@naver.com (D.H.S.); baksan@ee.knu.ac.kr (H.-G.L.); 2 Department of Biomedical Engineering, Kyungpook National University Hospital, 130 Dongdeok-ro, Jung-gu, 41944 Daegu, Korea; E-Mail: seongkw@ee.knu.ac.kr; 3 Department of Mechanical Engineering, Stanford University, 496 Lomita Mall, 94305 CA, USA; E-Mails: pkg1@stanford.edu (P.G.); puria@stanford.edu (S.P.); 4 Department of Otorhinolaryngology-Head and Neck Surgery, School of Medicine, Kyungpook National University, 680 Gukchaebosang-ro, Jung-gu, 41944 Daegu, Korea; E-Mail: kylee@knu.ac.kr; 5 School of Electronics Engineering, College of IT Engineering, Kyungpook National University, 80 Daehakro, Buk-gu, 41566 Daegu, Korea

**Keywords:** fully implantable hearing devices, implantable microphone, trans-tympanic, ventilation tube, cadaveric experiments, cochlear implants

## Abstract

Fully implantable hearing devices (FIHDs) have been developed as a new technology to overcome the disadvantages of conventional acoustic hearing aids. The implantable microphones currently used in FIHDs, however, have difficulty achieving high sensitivity to environmental sounds, low sensitivity to body noise, and ease of implantation. In general, implantable microphones may be placed under the skin in the temporal bone region of the skull. In this situation, body noise picked up during mastication and touching can be significant, and the layer of skin and hair can both attenuate and distort sounds. The new approach presently proposed is a microphone implanted at the tympanic membrane. This method increases the microphone’s sensitivity by utilizing the pinna’s directionally dependent sound collection capabilities and the natural resonances of the ear canal. The sensitivity and insertion loss of this microphone were measured in human cadaveric specimens in the 0.1 to 16 kHz frequency range. In addition, the maximum stable gain due to feedback between the trans-tympanic microphone and a round-window-drive transducer, was measured. The results confirmed *in situ* high-performance capabilities of the proposed trans-tympanic microphone.

## 1. Introduction

Fully implantable hearing devices (FIHDs) have been developed to assist the hearing impaired [1,2,3,4,5,6,7]. However, most FIHDs are still impractical because reliable, high-performance implantable microphones, which are crucial for FIHDs, have yet to be realized. Implantable microphones provide the input to FIHDs, and therefore have many requirements, such as high sensitivity, ease of implantation, clinical safety, bio-compatibility, and insensitivity to body noise and feedback.

**Figure 1:**
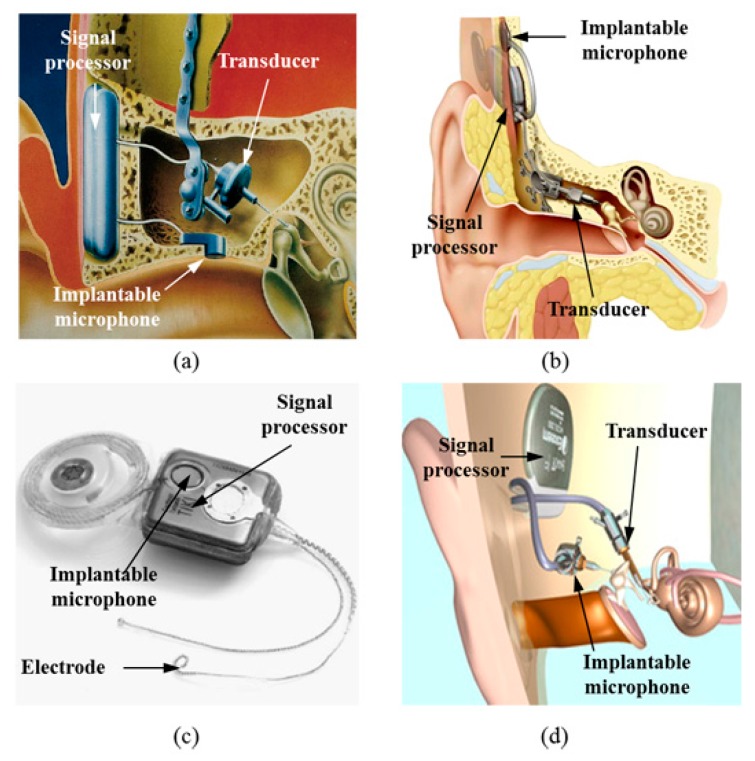
Microphone technologies used for fully implantable hearing aids: (**a**) TICA (Implex, Munich, Germany) [9]; (**b**) Carina (Cochlear, Sydney, Australia) [5]; (**c**) TIKI (Cochlear, Sydney, Australia) [4]; (**d**) Esteem (Envoy, Saint Paul, MN, USA) [5].

Many studies have been conducted on different types of FIHD microphones (Figure 1), including Totally Implantable Communication Assistance (TICA, Implex, Munich, Germany) [8,9], Carina (Cochlear, Sydney, Australia ) [5,10,11,12], Totally Implantable Cochlear Implant (TICI/TIKI, Cochlear, Sydney, Australia) [4,13,14] and Esteem (Envoy, Saint Paul, MN, USA) [5,15,16,17]. Carina and TIKI are typically implanted under the skin at the temporal bone, while TICA and Esteem are implanted in the ear canal or the ossicular chain, respectively. Each method has its own advantages and disadvantages [18,19,20]. Implanting the microphone under the skin at the temporal bone, such as for Carina and TIKI, gives rise to potential problems. These include significant body noise pickup during mastication, sound attenuation, and distortion from head touching due to the skin’s sound-filtering effect, and vulnerability to damage as a result of external impacts. Positioning the microphone in the ear canal or the middle ear, like for Esteem and TICA, results in less pickup of body noise and less sound attenuation. However, this method introduces an inherent feedback problem between the sound source and the implanted microphone. The TICA system was reported to be discontinued due to feedback problems between the middle-ear vibrator and the implantable microphone, as well as its extrusion into the ear canal from its original position [21]. For the reasons listed above, these types of implantable microphones have limited the commercial success of implantable hearing aids. To date, Esteem has been implanted in about 900 patients. On the other hand, Carina, TICA, and TIKI have been implanted in only 110, 19, and 3 patients, respectively [4,11,21,22]. Thus, new implantable microphone designs that can overcome the problems of existing designs are needed.

In this paper, we propose a new implantable microphone using a trans-tympanic ventilation tube in order to enhance the sensitivity and safety and to mitigate sound feedback from the middle-ear transducer. Because ventilation tubes are extensively used in otitis media treatments, their long-term safety has already been demonstrated [23]. The ultimate aim of the proposed method is to install all the components in the ventilation tube. However, in order to verify the feasibility of the proposed trans-tympanic microphone, the ventilation tube is connected to a microphone core that is located in the middle-ear wall via a flexible sound tube. The sensitivity of the trans-tympanic microphone, implanted in human cadaveric temporal bones, was measured and evaluated using acoustic measurements and laser Doppler vibrometry (LDV).

## 2. Conventional Implantable Microphones and the New Approach

Implantable microphones for FIHDs must have high sensitivity, a flat response, insensitivity to body noise, and ease of implantation during surgery. Figure 1 shows various types of FIHDs. As shown in Figure 1a, a microphone is implanted below the thin ear canal skin. Despite the fact that the skin has a thickness of only a few millimeters, it causes significant sensitivity degradation at high frequencies. In addition, the implanted microphones can sometimes extrude out of the skin. The frequency response of the implantable microphone in Figure 1a is relatively flat and has low sensitivity, because its diameter is restricted to 5 mm. 

A typical microphone implanted under the skin of the temporal bone is shown in Figure 1b. The mastoid area is wider than the ear canal, and this enables the installation of a microphone with a larger surface area. Therefore, the sensitivity is higher than that of the ear-canal implant. However, due to the filtering effect of the thick mastoid skin, severe degradation of sensitivity is unavoidable, especially in high-frequency regions. The low sensitivity of the microphone in high-frequency regions can decrease the system bandwidth and reduce hearing of target speech in the presence of spatially separated noise maskers [24]. Overall, problems associated with this type of microphone, such as body-noise pickup during mastication and head touching are unavoidable since it is implanted on the skull. Figure 1c shows another example of a mastoid-implant microphone. This is very similar to the microphone shown in Figure 1b, with the difference being that the microphone is embedded in the processor body, which restricts the microphone size. Thus, the microphone has a lower sensitivity in the audible frequency range due to the thick mastoid skin, and retains the disadvantages of body-noise pickup [4]. Figure 1d shows a microphone that measures ossicular vibration using a piezoelectric sensor attached on the malleus head. The microphone implanted in the middle-ear cavity will be more insensitive to body noise, because the acoustic sensor is located at the medial side of the tympanic membrane. The sensitivity of the microphone is fairly good, since it uses the natural auditory pathway, such as the resonance of the ear canal and the sound collection from the pinna. Despite the good sensitivity characteristics of this microphone, there is a high possibility of feedback because both the microphone and vibrator are part of the coupled ossicular chain. Therefore, a disarticulation of the incudo-stapedial joint is needed, which causes maximal conductive hearing loss and has the undesired effect of significantly reducing any residual hearing in the patient. Surgical placement is also difficult because the microphone’s performance is very dependent on the coupling between the piezo-sensor and the malleus head. 

**Figure 2:**
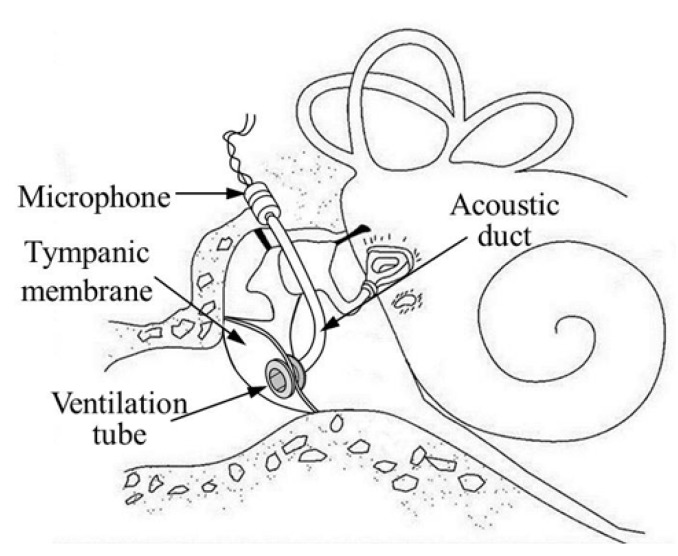
Conceptual diagram of the proposed trans-tympanic implantable microphone using a ventilation tube.

Additional studies similar to the one depicted in Figure 1d have been suggested, but with the position of the microphone at the umbo rather than the malleus head [25,26,27,28,29,30]. These studies tried to collect sound by using the inertial force of the tympanic membrane. Ko *et al.* (2008) designed a microphone that is directly mounted on the umbo using a micro-electromechanical system (MEMS) capacitive sensor [27,28,29]. The diaphragm, which makes up one side of the two-layer capacitive sensor, is coupled with the umbo. Sound pressure coming from the ear canal vibrates the tympanic membrane and umbo. The capacitance is modulated due to inertial forces varying the distance between the diaphragm and substrate. Unfortunately, this scheme has low-frequency attenuation due to loading of the sensor mass on the umbo. Young *et al.* (2012) improved this method by using a multilayer MEMS capacitive sensor to improve the sensitivity, but the problem of mass loading still remained [28,29]. Similarly, Yip *et al.* (2015) suggested a microphone using a piezoelectric sensor with one end attached to the umbo and the other clamped on the middle-ear wall like a cantilever [30]. The sensor detects the sound-driver motion of the umbo. Because this type of microphone is similar to Esteem, the feedback problem is unavoidable. Therefore, Yip’s microphone was just used for a cochlear-implant system. In another scheme, an ultra-miniature accelerometer (one of smallest ever fabricated) was developed using piezo-resist MEMS fabrication methods [31]. The accelerometer was evaluated in human cadaveric temporal bones and was shown to have a noise floor of approximately 60 dB SPL, which is high for use in an implantable hearing-aid application. In this paper, we propose a new structure for the microphone in order to overcome the disadvantages of the conventional types. The suggested implantable microphone, as shown in Figure 2, consists of a ventilation tube (Paparella type I, Medtronic Inc., Dublin, Ireland), a 76-mm-long acoustic duct (ER7-14C, Etymotic Inc., Elk Grove Village, IL, USA), and an electret microphone (EM, FG-23329-C05, Knowles Electronics, Itasca, IL, USA). The ventilation tube, commonly used in otolaryngology, is implanted in the tympanic membrane, and the sound collected from the ventilation tube is transmitted through the acoustic duct to the small electret microphone implanted in the middle-ear cavity. This scheme increases the microphone’s sensitivity in several ways, such as by utilizing the pinna’s sound collection and the ear canal resonance. In addition to FIHDs, the proposed trans-tympanic microphone can also be used in the future design of fully implantable cochlear implants. 

## 3. Measurement Methods

### 3.1. Temporal-Bone Preparation

In this paper, the performance of the proposed trans-tympanic microphone was evaluated in human cadaveric temporal bones (N = 3). Feedback characteristics were also investigated when the microphone was used with an FIHD with a vibrational transducer output applied at the round-window membrane. All temporal bones used in the experiments were obtained from human bodies within 48 h of death and were frozen and stored after extraction (Anatomy Gifts Registry, Hanover, MD, USA). Prior to performing the experiments, it was confirmed that the temporal bones had the characteristics of normal auditory pathways. The middle-ear cavities of all temporal bones were opened via the mastoid bone using a surgical drill to obtain access to the stapes, and the ear canal was completely removed. It was then replaced by an artificial ear canal 30 mm long comprised of a cylindrical plastic tube. The drilled bone was wrapped with clay in order to seal in moisture and minimize changes in mechanical properties throughout the duration of the measurements. The probe tube from a reference microphone system (ER-7C, Etymotic Research Inc., Elk Grove Village, IL, USA) was placed at the entrance of the artificial ear canal in order to calibrate the sound pressure. All temporal-bone measurements were performed at the Stanford OtoBiomechanics Laboratory.

### 3.2. Measurements and Data Acquisition

**Figure 3:**
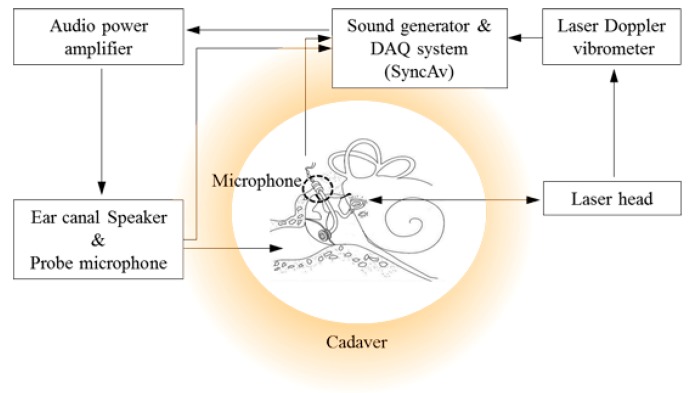
Block diagram of cadaveric temporal bone experiments for *in situ* verification of the proposed trans-tympanic microphone performance.

A high-frequency tweeter (NE25VTS, Tymphany HK Ltd, Sausalito, CA, USA) with a closed-field sound output was coupled to the artificial ear canal. The vibration velocity of the stapes was measured using a laser Doppler vibrometer (HLV-1000, Polytec GmbH, Irvine, CA, USA) focused on reflective beads placed at the center of the stapes footplate. SyncAv v0.26 (Stanford University, Stanford, CA, USA), running on a National Instruments PXI 4461 DAQ (National Instruments, Austin, TX, USA), was used to generate pure tones from 0.1 to 16 kHz with 74 logarithmically spaced steps. The ear-canal pressure was equalized to approximately 94 dB SPL using the SyncAv “Stimulus EQ” function. Simultaneous with the generated stimulus, SyncAv recorded the stapes velocity and ear-canal sound pressure and performed synchronous averaging to reduce noise. The sampling rate, FFT length, and number of averages were 96 kHz, 4096, and 10, respectively. The experimental setup for measuring the characteristics of the proposed trans-tympanic microphone is shown in Figure 3.

## 4. Results

### 4.1. Results of the Trans-Tympanic Microphone Experiment

**Figure 4:**
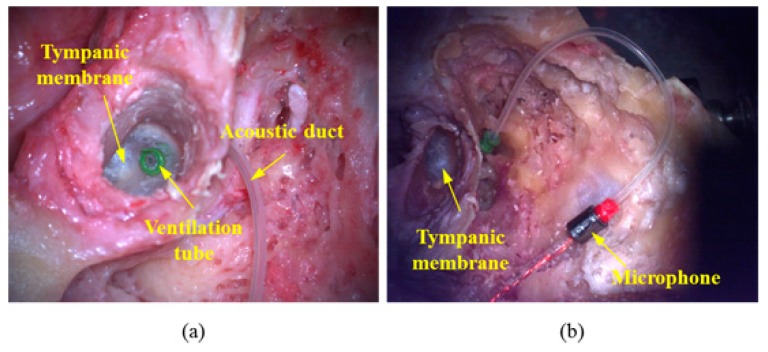
Photograph of installed microphone at the tympanic membrane and middle-ear cavity. (**a**) Front view of the tympanic membrane with installed ventilation tube; (**b**) rear view of the tympanic membrane with tube connecting the ventilation tube and microphone (in this view, the ventilation tube has not yet been placed through the tympanic membrane).

The proposed trans-tympanic microphone consists of a ventilation tube, acoustic duct, and electret microphone. This assembly was installed in the tympanic membrane and middle-ear cavity as shown *in situ* in Figure 4. Prior to the performance evaluation of the microphone, the baseline stapes-footplate velocity of each temporal bone was measured in response to an approximately constant ear-canal input pressure of 94 dB SPL from 0.1 to 16 kHz.

**Figure 5:**
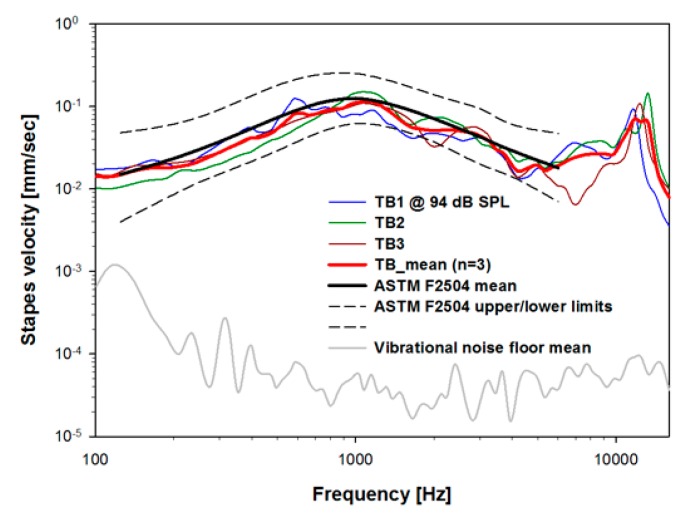
Stapes velocity for approximately 94 dB SPL sound drive at the entrance of the artificial ear canal in TB1-3 and their mean. The ASTM F2504 standard response is shown for comparison. The noise floor was measured without a sound drive.

Figure 5 shows the vibration characteristics of the stapes of the individual temporal bones (blue, green, and brown solid lines) and the mean value (red bold line) of these measurements. Above approximately 200 Hz, the stapes-velocity noise floor (gray solid line), measured without a sound stimulus, was 40 dB or more lower than the mean stapes velocity. The resonance frequency of stapes vibration at 1 kHz had a maximum value of approximately 0.1 mm per sec. In addition, this figure shows the characteristics of normal stapes vibration (ASTM F2504, black solid and dashed lines), with bilateral symmetry and a −20 dB per decade decrease from 1 to 10 kHz. 

**Figure 6:**
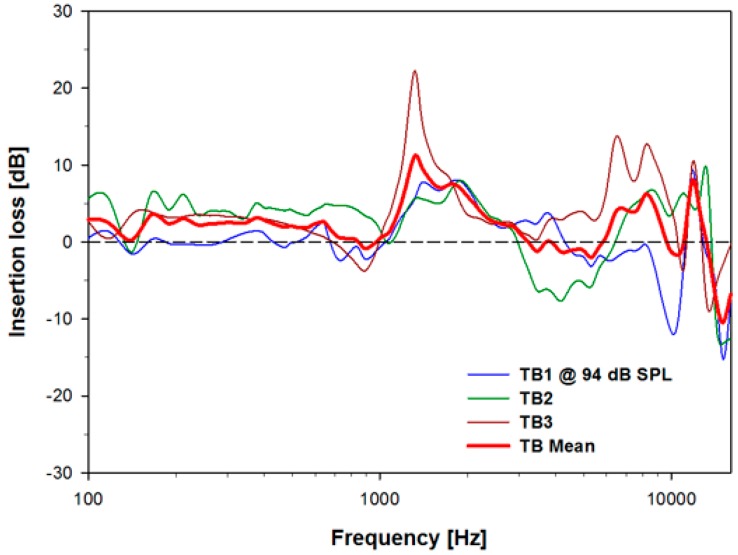
Insertion loss due to placement of the microphone on the tympanic membrane, which is the difference in dB between the stapes velocity before and after placement of the ventilation tube on the tympanic membrane, for TB1-3 and the mean value.

Since the trans-tympanic microphone is placed in the auditory pathway, the stiffness and 100 mg total mass of the implanted microphone may have a negative effect on residual hearing. Therefore, the insertion loss elicited from variations in stapes velocity before and after implantation was measured. Figure 6 shows the insertion losses of the individuals TB1–TB3 (blue, green, and brown solid lines), and the mean value (red bold line) of these measurements. The mass of the trans-tympanic tube and tube stiffness elicited an average reduction of residual hearing across all frequencies of 2.1 dB and upper and lower limits of the mean of approximately ±10 dB on residual hearing for the three temporal bones used in this experiment.

**Figure 7:**
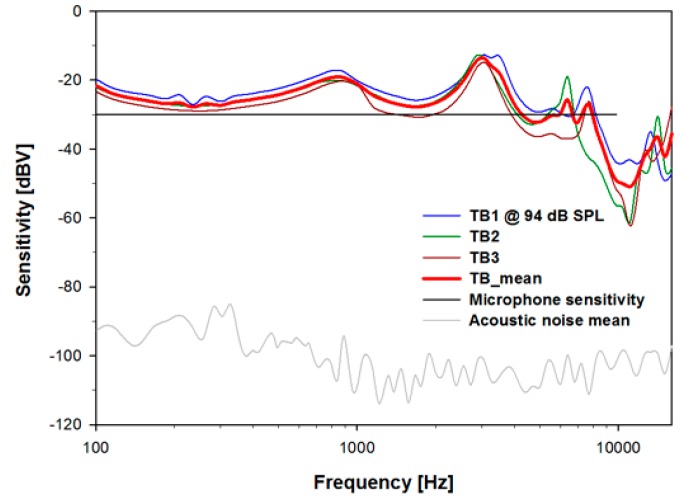
Voltage sensitivity of the *in situ* trans-tympanic microphone with 94 dB SPL sound pressure at the entrance of the ear canal for TB1–3 and their mean. The average noise floor is typically 60–70 dB lower.

To determine the characteristics of sound collection of the trans-tympanic microphone, we measured the output signal after implanting the proposed microphone at the tympanic membrane with a 94 dB SPL (1 Pascal) stimulus at the entrance of the ear canal. Figure 7 shows the results elicited from the three temporal bones. The acoustic noise floor of the implanted microphone, which was measured without sound stimulus, was typically −100 to −90 dBV. The microphones had a flat sensitivity of −30 dBV per Pa in air, thus the measured noise floor was equivalent to 24 to 34 dB SPL (per 93.75 Hz bin). 

There are two quarter-wavelength tube resonances at approximately 0.85 and 3 kHz. The 3 kHz resonance is due to the 30 mm artificial ear canal tube and the 0.85 kHz resonance is due to the length of total acoustic pathway including the 76 mm acoustic duct. The average *in situ* sensitivity (Figure 7d, red bold line) was approximately −26 dBV per Pa in the 0.1–10 kHz range. Considering that current microphones designed for implantation under the skin show acoustic attenuation of approximately −40 dB per decade above 1 kHz [19], the results in Figure 7 demonstrate that the tympanic membrane is a sensitive location for an implantable microphone with respect to sound collection.

### 4.2. Acoustic Feedback for the Trans-Tympanic Microphone with Round-Window Drive

Because the transducer and the proposed microphone are located on the same acousto-mechanical pathway, one can expect that acoustic feedback may be significant when the middle ear is driven. To test this, a new round-window-drive transducer we recently designed to avoid the mass-loading effect on the middle ear was used [32]. 

To measure the maximum stable gain (MSG) between the trans-tympanic microphone and the middle-ear transducer, we measured the electrical outputs of the microphones after their implantation at the tympanic membrane of the same three temporal bones (TB1–3). Mechanical stimulation was applied to the round window (RW), and the resulting stapes velocity and feedback pressure in the ear cancel were then scaled so that the stapes velocity matched that from a 94 dB SPL (1 Pa) acoustic stimulation at the ear-canal entrance. From these measurements the MSG in dB was calculated using the formula:

(1) $$\text{MSG}=20\times \text{log}({\text{V}}_{\text{sd}}/{\text{V}}_{\text{f}})$$

where V_sd_ is the sound-driven microphone voltage and V_f_ is the microphone voltage due to feedback pressure generated by the movement of the tympanic membrane as a result of the backward stimulation of the ossicular chain driven at the RW. For both the ear canal forward sound and reverse RW drives the stapes velocity was driven by the same amount to produce an equivalent pressure of 1 Pa.

**Figure 8:**
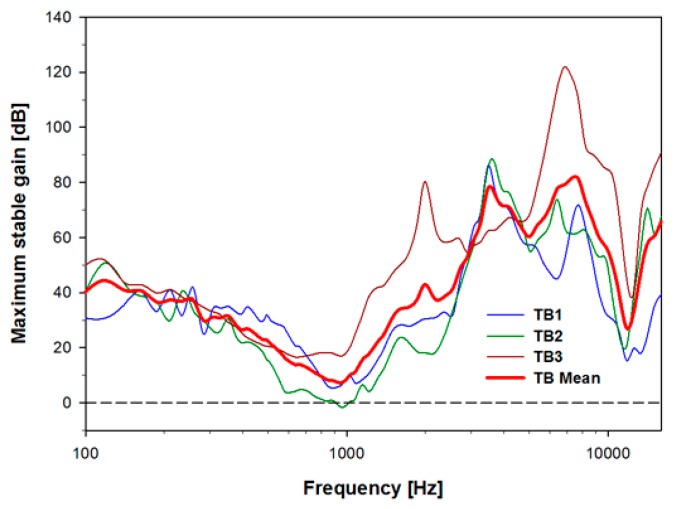
Maximum stable gain (MSG) of the trans-tympanic microphone when the round window was driven by a vibrational transducer at a level equivalent to 94 dB SPL at the ear-canal entrance, for TB1–3 and the mean value.

As shown in Figure 8, the three ear MSG mean rolled off with a slope of about −12 dB per octave below 1 kHz, and rose approximately +18 dB per octave in the 1–8 kHz range. The average gain across frequencies is 41 dB. The minimum gain is about 7 dB near 1 kHz, while the maximum gain is about 80 dB near 7 kHz. A feedback control circuit can be used to improve the MSG, particularly in the 1 kHz mid-frequency region, which can reduce the response of the feedback by approximately 12 dB [33]. Considering the development trends of feedback-cancellation algorithms, the proposed microphone will be applicable for FIHDs, especially because, as most hearing loss occurs at high frequencies, significant gain is typically not required below approximately 1.5 kHz.

## 5. Discussion and Conclusions

These measurements demonstrate that the proposed trans-tympanic microphone has minimal impact on the normal function of the tympanic membrane (Figure 6), has high sensitivity (Figure 7), and has a high gain margin outside of the middle-ear resonance frequency of approximately 1 kHz (Figure 8). It is therefore a promising device for use with next-generation FIHDs.

There are three potential issues that may arise when using this device *in vivo*. First, there is a concern that cerumen may block the microphone and prevent normal sound collection. However, cerumen is only produced in the lateral third of the ear canal, migrates laterally, and removes itself from the ear canal [34,35,36]. It is therefore unlikely that the microphone would become blocked unless the cerumen were impacted, which can be prevented through routine medical care [37]. Second, it is possible that the ventilation tube would migrate with the epithelium toward the ear canal, or in rare cases, medially toward the umbo [38]. This could cause the tube to fall into the ear canal or middle-ear cavity, which would require revision surgery. This can be addressed by tethering the ventilation tube to prevent movement. Finally, steps will need to be taken to prevent fluid from entering the ventilation tube and negatively affecting microphone sensitivity. Future designs could incorporate a membrane or filter at the tip of the ventilation tube. Further studies will be required to evaluate the *in vivo* performance of the microphone.

Practical issues relevant to FIHD microphones were reviewed in this paper. To overcome the limitations of previous implantable microphones, a prototype trans-tympanic microphone was proposed. The structure of this device consists of a ventilation tube installed in the tympanic membrane and a connected acoustic duct extending into the middle-ear cavity with an electret microphone at the end. The proposed microphone has several potential benefits, such as outstanding sound collection, relatively simple surgery, and safety from external impacts. The feasibility of the proposed microphone was verified based on measurements and analysis of the device as installed in previously frozen cadaveric temporal bones.
